# Spotlight on the trophic response of crop plants under shade temporal fluctuation

**DOI:** 10.1017/qpb.2026.10049

**Published:** 2026-06-24

**Authors:** Nicolas Dusart

**Affiliations:** INRAE, URP3F, 86600 Lusignan, France

**Keywords:** carbon, crops, light, shade, yield

## Abstract

The emergence of complex cropping systems involving dual land use raises new questions regarding plant growth under intermittent shade conditions. Recent meta-analyses have investigated the dose–response relationships of several plant traits to light availability, highlighting the strong interest in understanding plant responses to light temporal variation. However, these studies also reveal significant gaps in our knowledge, particularly concerning plant responses to low-light conditions and the determinants of crop yield under shade. In this context, physiological mechanisms related to photosynthesis, growth, reproduction and, more broadly, carbon allocation appear to play a central role. This article emphasizes the major implications of carbon allocation, storage and use under prolonged and fluctuating shaded conditions for crop production.

## Graphical abstract

**Figure:**
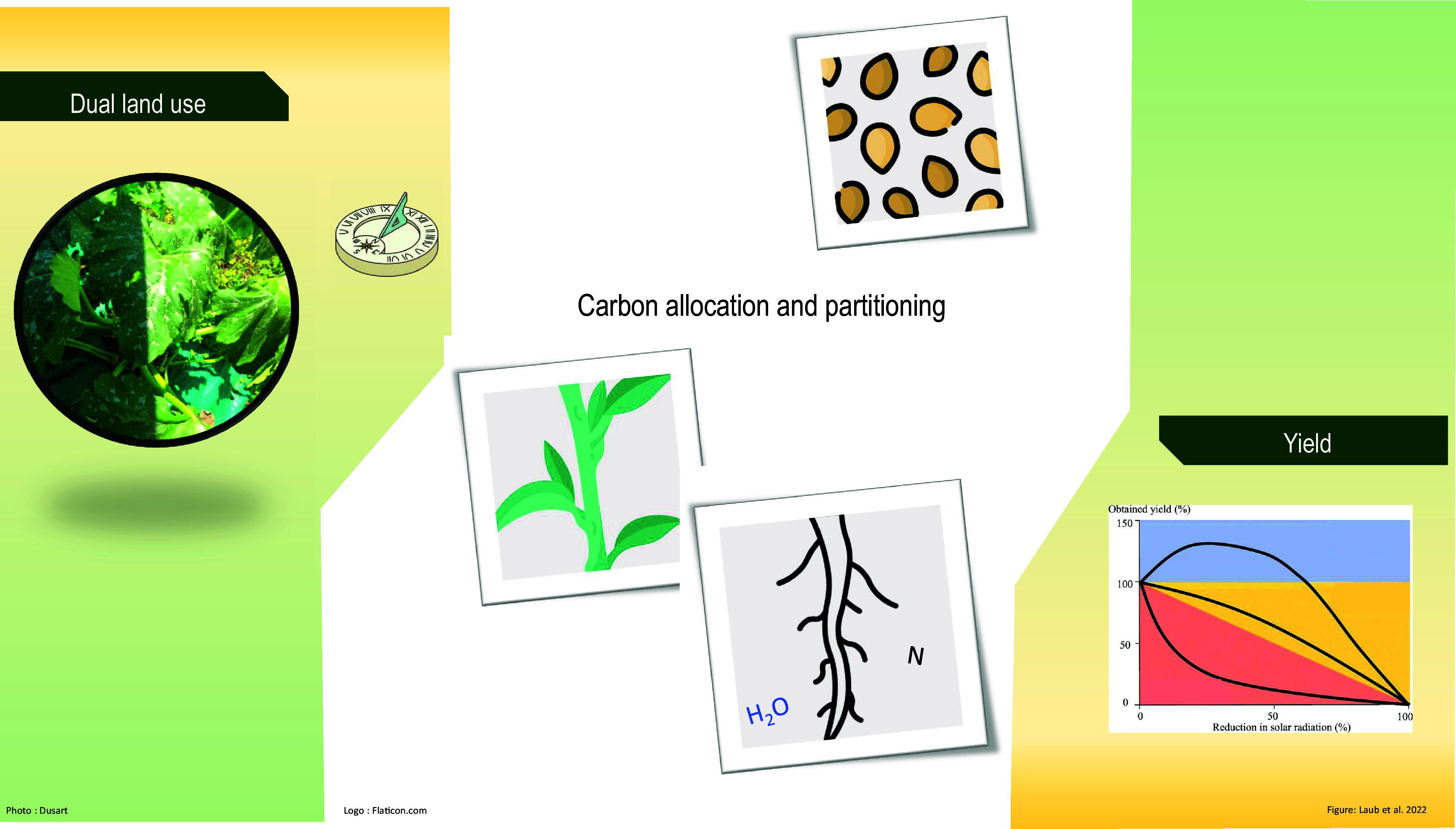

For several years now, under the combined influence of climate change and economic needs, dual land use systems have been promoted (Kala, 2025; Malézieux et al., 2009; Toledo & Scognamiglio, 2021). These systems incorporate service plants for intercropping (growing multiple crops together at the same time), relay cropping (introducing a second crop before the first one is harvested), agroforestry or energy production through solar panel installation (Figure 1). During domestication and breeding, crop plants were selected under light regimes characterized by full sunlight and dense plant stands (Donald, 1968). Recent research has shifted towards examining crop responses to shade, particularly within complex cropping systems. This paradigm shift requires careful consideration of the characteristics of the whole microclimate and its impact on plant physiological responses. The development of these systems has generated and will generate a large amount of data for quantitative analysis.

**Figure 1.**
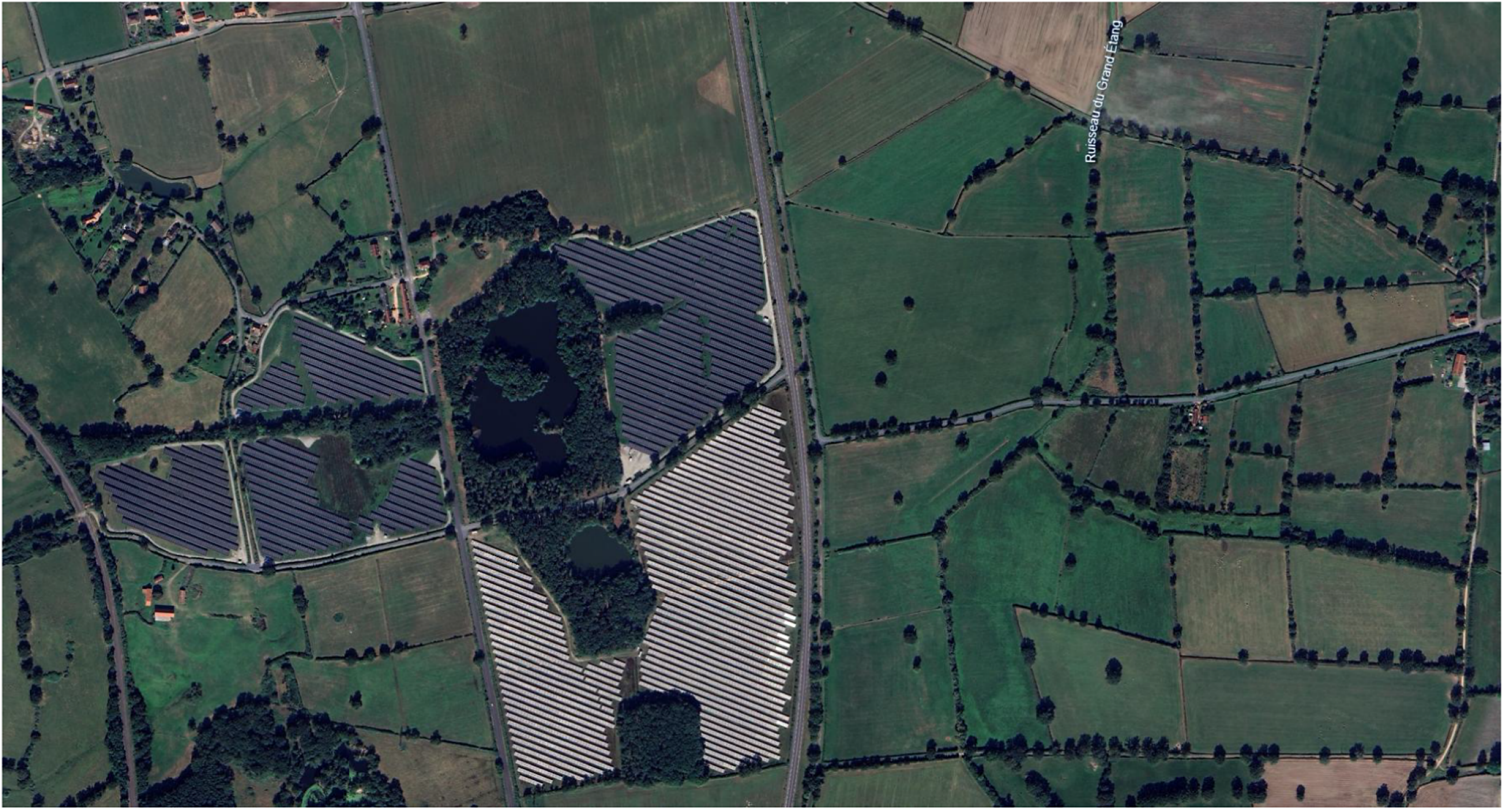
Agricultural landscape incorporating a photovoltaic power station, forest, livestock farming and arable crops, separated by hedgerows. (46°18′49″ N, 2°44′39″ E). Source: Google Earth, © Google, September 16, 2024. Figure 1. long description.

Behind the notion of shading lies a multitude of different conditions, linked in particular to spatial and temporal fluctuations depending on the type of system. On a spatial scale, starting with the plant, the architecture and morphogenesis of leaves influence light interception (Niinemets & Valladares, 2004). The light quality is also modified by the plant canopy, which results in a change in the red/far-red (R/FR) ratio, probably the most studied photomorphogenic signal in plants (Ballaré & Pierik, 2017). Within the stand, competition between neighbouring plants creates heterogeneities in canopy cover (Perez et al., 2024), which can be found in agroforestry and with photovoltaic panels on another spatial scale. On a temporal scale, shading can be linked to different time steps: (i) infra-hourly, such as sunflecks (brief bursts of light through canopy gaps) requiring rapid photosynthetic induction (Durand et al., 2021); (ii) daily, resulting from solar trajectory and light interception by neighbouring obstacles (plant, structure, cloud, etc.); and (iii) seasonal, depending on the sun’s path, which triggers a long-term physiological acclimation. In this respect, agroforestry and photovoltaic panels (excluding automated shade structures) differ: tree shading varies throughout the seasons according to their phenology, which can generate competition for soil resources, while photovoltaic panels, which are fixed, undergo variations linked solely to the seasonal path of the sun, without competition for other resources. This difference requires the shade to be carefully characterized according to the systems under study.

The characterization of shade is intrinsically linked to classical light measurements, ranging from the overall energy budget (W.m^-2^) to the assessment of photosynthetic photon flux density (PPFD, μmol.m^-2^s^-1^). Considering the total energy balance (short and long waves) allows a more complete representation of light impact on temperature, transpiration and photosynthesis. In addition, specific wavelengths, such as ultraviolet radiation or the R/FR ratio, can strongly influence plant development and may also be considered for their photomorphogenic effects (for review, see Ballaré & Pierik, 2017). However, most studies on light intensity focus on the 400–700 nm waveband, which corresponds to photosynthetically active radiation and is integrated into PPFD measurements. To account for the temporal variability of light over the day, PPFD is commonly integrated into the daily light integral (DLI, mol·m^-2^·d^-1^). Numerous plant traits have been shown to correlate more strongly with DLI than with instantaneous PPFD values measured at a specific time point (Monteith, 1977; Niinemets et al., 2015).

Regarding the timing of shade exposure and sun–shade transitions during the day, multiple sun–shade transitions can occur (Varella et al., 2011; Yajima et al., 2023). These transitions depend on the systems under study and on the orientation of structures that generate shading. A recent work on the timing of shade exposure (morning vs. afternoon) under similar daily light integrals (DLIs) has revealed differences in biomass accumulation among pasture species (Owston, 2025). This challenges the general assumption that shade tolerance can be predicted solely from plant responses to uniform reductions in light. Gas-exchange kinetic properties, which determine how rapidly photosynthesis reaches full capacity following shade-to-sun transitions, are therefore critical (Durand et al., 2022; Pearcy & Way, 2012; Porcar-Castell & Palmroth, 2012). These processes remain underexplored in agrivoltaic and agroforestry systems, raising new and challenging questions. How does intermittent shading modulate plant responses to low light, particularly carbon assimilation and integrated physiological responses? Do identical low DLI values but contrasting shading interruption patterns elicit similar plant responses? To address these questions, it is essential to place short-term responses within a broader plant response to light.

In the meta-analysis of Poorter et al. (2019), the authors explored a large database of plant traits related to anatomy, morphology, physiology, growth or reproduction expressed as dose–response curves against DLI. Approximately 40% of the 70 variables considered show a saturating response to light. At high light conditions, some responses are probably associated with oxidative stress or metabolic limitation. Interestingly, their approach enables us to identify some gaps in our knowledge. No differences in plasticity were detected between shade-tolerant and light-demanding species. However, higher leaf mass area and/or tissue density in shade-tolerant species supports the stress-tolerance hypothesis, that is, a greater allocation of resources to defence and storage. Although their data did not provide evidence of carbon maximization per time unit, a positive carbon gain is, of course, essential in the long term. The underlying insight into the regulation of long-term responses of phenotypic traits is poor, and this is particularly true for C limitation at low light. It seems to me that this specific area is of particular concern for perennial plants in shade systems. Plant reproduction is also an understudied process in response to DLI, mainly because the time required for seed or fruit production often exceeds the duration of most experiments. Nevertheless, it emerges as one of the most plastic traits (Poorter et al., 2019). This process is of particular interest because of its crucial role in yield determination for numerous crops, including wheat, berries and fruit trees. To go further, the authors also raise an important ecological question: “What exactly causes light-demanding species to succumb under low light conditions, and why do shade-tolerant species perform so much better?” (Poorter et al., 2019). Building on this, I would add the question of whether carbon reserves and their allocation play a central role in plant responses to shading, particularly in their interactions with other biotic and abiotic environmental factors. These aspects warrant a dedicated discussion of plant responses to multiple stresses. In the present *Insight*, it will be kept brief. Studies have highlighted functional trade-offs between light and drought tolerance (Niinemets & Valladares, 2006). Beyond this trade-off, modifications of the energy balance directly influence organ temperature and plant water use. Agrivoltaic systems illustrate how structural shading modifies plant energy balance, reducing thermal stress and ultimately buffering drought impacts (Barron-Gafford et al., 2019).

In a recent meta-analysis on crop yield responses to shading, Laub et al. (2022) hypothesized that, across crop types, yield responses to shading differ significantly. They proposed to classify crops into three categories: shade-benefiting, shade-tolerant and shade-susceptible. Shade-benefiting crops are defined as those exhibiting an increase in yield under low levels of shading, with yield declines occurring only at higher shading intensities, a pattern reminiscent of hormesis (Calabrese & Blain, 2009). The results of Laub et al. (2022) indicate that crop responses to shading vary by type. Berries, fruits and fruity vegetables can benefit from moderate shade, up to ~40% reduction in solar radiation (RSR). Forages, leafy vegetables and tuber/root crops show limited yield losses under low shading (20% RSR), whereas maize and grain legumes are highly shade-sensitive. This type of response curve could be useful for determining the association and shading level in a dual-land use system. Beyond the observed yield responses to shading, a critical question concerns the parameters used to define and quantify yield. Does yield primarily reflect total biomass production, fruit yield or the accumulation of storage organs? The French idiom “to compare cabbages and carrots” highlights this issue, as yield may be characterized by fundamentally different biomass compartments, vegetative versus reproductive tissues, leaves versus fruits or below-ground storage organs and expressed in different units, such as fresh mass versus dry mass. These distinctions may partly explain differences in yield losses among crops, particularly between those whose yield is expressed as dry matter and those measured as fresh biomass. Yet, ultimately, they represent comparable physiological and functional responses across crop plants.

From my point of view, carbon allocation and partitioning at the whole plant level are central to this question. Taking into account the developmental stage, carbon allocation for growth or reproduction seems essential. For instance, a decline in the yield of maize under shading has been linked to alterations in the source-sink allocation (Liang et al., 2025). For perennial plants, comparing shade-tolerant versus light-demanding tree seedling revealed a partitioning difference between compartments, with the shade-tolerant species having more height growth and storage (Giertych et al., 2015). Shading duration significantly influenced mixed pasture yield, which declined by about 10% after 3 months and by up to 50% after 12 months at 80% shade (Dodd et al., 2005). In addition, shading strongly altered pasture species composition, reducing legume abundance while favouring grasses (Dodd et al., 2005). Long-term effects are largely missing from existing studies. In both meta-analyses (Laub et al., 2022; Poorter et al., 2019), most studies relied on data collected over one or two years or were based on short-term controlled experiments. As a result, the long-term effects of shading remain poorly understood. Only a limited number of studies have investigated the effects of shading over multiple years. Atlan et al. (2015) showed that 2 years of shading induced a strong but reversible reduction in flowering in common gorse. This raised questions around the long-term evolution of perennial crops (fruit trees, grapes, pasture, etc.) in these systems. Otherwise, a 2-year agrivoltaic experiment on alfalfa revealed variable yield responses between years, likely linked to differences in water availability (Edouard et al., 2023). Shading decreased the yield by itself, but when combined with drought, it mitigated the effect of the water deficit on the yield (Edouard et al., 2023). Even if shade reduces yield, it may counteract the effects of stochastic extreme events (e.g., drought and heat wave), making the agroecosystem more robust. But long-term experiments are needed to understand the effects of hazards and shade.

The optimal partitioning theory (also referred to as the functional equilibrium hypothesis) predicts that plants adjust biomass allocation toward the organ responsible for acquiring the most limiting resource in order to maximize growth (Bloom et al., 1985; Poorter et al., 2012). In low-light conditions, aboveground biomass is favoured at the expense of belowground parts. However, such a shift may reduce the capacity of the plant to acquire other essential resources, such as nitrogen or water. Moreover, an increase in the leaf area or the aboveground biomass may intensify competition for light within the stand, potentially constraining overall growth. Golan et al. (2024) proposed a conceptual framework for investigating the genetic determinants that underlie resource allocation strategies in wheat under canopy shade conditions. This study suggests that agroecological practices may drive the selection of less competitive individual plants, thereby enhancing overall population yield (Donald, 1968; Golan et al., 2024). In the wheat case, an ideotype that prioritizes grain filling by sacrificing resources that would be used for competitive structures (such as leaves and stems) results from a trade-off between plant density/competition and production. This could benefit from an intercropping system, for example, cereal and legume.

Light intensity and shading generate dose–response curves for many physiological parameters. By definition, there is an optimal light level for crops, which can vary among species and across spatial contexts. Precisely determining which parameters control plasticity and, ultimately, yield is of primary importance in this expanding field of research. Current research highlights the lack of data for large response amplitudes, from low to high light, particularly under fluctuating low-light conditions. Consequently, future research should explore plant responses to light across a much wider range of intensities and various temporal fluctuations. Regarding intermittent shading, experiments comparing natural fluctuating light regimes with traditional square light have revealed a strong epigenetic regulation in *Arabidopsis thaliana* (Emmerson et al., 2025). These changes are associated with the regulation of genes involved in photosynthetic efficiency. A more recent work on rice has shown that intermittent shading leads to greater yield losses (~5–10% more than those caused by a simple reduction in light intensity), likely due to a decreased photosynthetic efficiency and an amplification of stress avoidance responses (Lescroart et al., 2026).

We should prioritize the characterization of daily and infra-hourly light patterns in different systems. Light quantitative data from various systems, notably agrivoltaic ones, could be used. This will also involve unravelling the effects of this intermittency on gas exchange dynamics, which could ultimately impact plant development. Whole-plant carbon balance plays a central role in these processes, and for perennial crops, long-term studies are needed to assess carbon starvation and plant mortality under shading. Finally, the system should be considered as a whole: there may be trade-offs between optimizing crop yield through shade manipulation and maximizing the overall economic performance of the system. Accepting a short-term or local yield loss may, in some cases, provide greater benefits by increasing system resilience to environmental stresses (pest, drought, heat wave, freezing events, etc.).

## Data Availability

No data or code were developed for this manuscript.

## Long descriptions

### Figure 1. Long description

The aerial view is centered on a large P V power station composed of several distinct clusters of dark solar panels. At the heart of the installation is a dense, dark green forest patch containing a small body of water. To the East of the solar clusters, the landscape transitions into a mosaic of bright green agricultural fields and livestock pastures. These fields are strictly partitioned by a network of dark green hedgerows and narrow tree lines, creating a grid-like pattern. A prominent North-South road bisects the image, separating the primary solar installation from the eastern arable lands. In the Northwest and Southwest corners, small clusters of farm buildings and residential structures are visible, connected by thin access roads. The Ruisseau du Grand Etang stream is labeled in the Northeast quadrant, cutting through the hedgerow-lined fields.

Navigate back to Figure 1..
