# Supplementary material for: Spotlight on the trophic response of crop plants under shade temporal fluctuation
Source: Quant Plant Biol. 2026 Jun 24;7:e14. doi: 10.1017/qpb.2026.10049 (PMC13430473; doi:10.1017/qpb.2026.10049)
Supplement: Peer review history

# Reviewer report: Review: Spotlight on the trophic response of crop plants under shade temporal fluctuation — R0/PR2

**DOI:** 10.1017/qpb.2026.10049.pr2

**Authors:** Robyn Emmerson

**Affiliations:** Biology, University of Oxford Life and Environmental Sciences Division, United Kingdom of Great Britain and Northern Ireland

This insight provides a concise overview of the literature relating to fluctuations in light/shade in agricultural systems and suggests how dual land use may impact this. The author discusses the factors that are linked to shading, including light intensity and timing of exposure, linking these to relevant plant physiological traits.

There are some instances where the intended meaning is unclear:

-“such as cloud cover affecting stomatal conductance (Vialet-Chabrand et al. 2017)”. This paper attributes some fluctuations in light to cloud cover, but does not directly attribute changes in stomatal conductance to changes in light caused by cloud cover. Maybe reword this to put less emphasis on the cloud cover and more on the diurnal fluctuations for a range of reasons.

-“For perennial plant, comparing tree seedling shade tolerant vs light demanding revealed”- do you mean shade tolerance vs light demanding varieties?

-“Shading duration significantly influenced pasture yield”- you later go on to mention legume abundance, is this the crop in the pasture studied?

-"This study suggests that agroecological practices may drive the selection of

less competitive individual plants, thereby enhancing overall population yield"- I think this would benefit from some further explanation, it is not currently clear to me how selection of less competitive plants could enhance yield. Do you mean that there has been selection for plants that compete less for resources e.g. light (so shade neighbours less), therefore reducing competition between individuals within the population and so increasing overall yield?

Additional comments:

- from the introductory paragraph, I was expecting the theme of dual land use to thread through the manuscript. It isn’t entirely clear how dual land use is impacting some of the parameters mentioned

Other than these, I found the insight enjoyable and thought-provoking

# Reviewer report: Review: Spotlight on the trophic response of crop plants under shade temporal fluctuation — R0/PR3

**DOI:** 10.1017/qpb.2026.10049.pr3

I enjoyed reading this perspective and I think the authors discuss a very important aspect on light (patterns and intensity) and plant growth.

One thing that could improve this manuscript would be to discuss a recent finding on how changes in light patters (fluctuating light vs square light) leads to more epigenetic changes (mainly in transposable elements) compared to changes in light intensity (low vs high); see PMID:40974118.

# Reviewer report: Review: Spotlight on the trophic response of crop plants under shade temporal fluctuation — R0/PR4

**DOI:** 10.1017/qpb.2026.10049.pr4

This is a nice insight paper offering a fresh perspective on plant responses to shade. However, the main message of the manuscript escapes me a bit: what should we prioritize exactly in terms of research to most efficiently advance our knowledge on crop growth under intermittent shade conditions? Maybe a conclusion paragraph would be useful. I also have a number of minor comments:

- Terminology such as relay cropping, intercropping should be defined at first use

- R/FR ratio should be spelled out at first use (it is spelled out later in the text)

- The manuscript could benefit from proofreading by a native English speaker. Given the lack of line numbers in the manuscript, it is difficult to list the errors, but they frequently involve using singular terms where plurals should be used.

- ‘… the authors explored a large database of plant traits related to anatomy, morphology, physiology growth or reproduction express as dose response curve against DLI’. I assume this should be ‘a large database of plant traits related to anatomy, morphology, physiology, growth or reproduction expressed as dose response curves against DLI’.

- ‘…across crop types, yield responses to shading differ significantly and can be classified into three categories…’ should be ‘…across crop types, yield responses to shading differ significantly and crop types can be classified into three categories…’.

- ‘up to approx. 40% reduction solar radiation (RSR)’ should be ‘up to approx. 40% reduction in solar radiation (RSR)’.

- ‘For perennial plant, comparing tree seedling shade tolerant vs light demanding revealed a partitioning difference between compartment with more height growth and storage for shade tolerant (Giertych et al. 2015).’ : I do not understand this sentence.

- ‘Similarly, a two-year agrivoltaic experiment on alfalfa revealed variable yield responses between years, likely linked to differences in water availability (Edouard et al. 2023).’ : how does this link to shading responses ?

- ‘Current research highlights the lack of data for large response amplitudes, particularly under low-light conditions.’ : what is meant with ‘large response amplitudes’ ?

# Editor report: Recommendation: Spotlight on the trophic response of crop plants under shade temporal fluctuation — R0/PR5

**DOI:** 10.1017/qpb.2026.10049.pr5

**Affiliations:** Biology, University of York, United Kingdom of Great Britain and Northern Ireland

Please see the minor revisions suggested by the review. Please especially consider addressing the question posed by the third reviewer: “ what should we prioritize exactly in terms of research to most efficiently advance our knowledge on crop growth under intermittent shade conditions?”

# Editor report: Decision: Spotlight on the trophic response of crop plants under shade temporal fluctuation — R0/PR6

**DOI:** 10.1017/qpb.2026.10049.pr6

**Affiliations:** RDP, ENS de Lyon, France

No accompanying comment.

# Reviewer report: Review: Spotlight on the trophic response of crop plants under shade temporal fluctuation — R1/PR8

**DOI:** 10.1017/qpb.2026.10049.pr8

**Authors:** Robyn Emmerson

**Affiliations:** Biology, University of Oxford Life and Environmental Sciences Division, United Kingdom of Great Britain and Northern Ireland

Thank you for your revisions, all my comments have been addressed. I have one very minor point; on line 165 you refer to the study utilising “chamber experiments” which is not the case. I suggest just removing the word “chamber” or replacing this with “controlled”/similar if you want to make the point that this was a laboratory experiment

# Reviewer report: Review: Spotlight on the trophic response of crop plants under shade temporal fluctuation — R1/PR9

**DOI:** 10.1017/qpb.2026.10049.pr9

The authors addressed my comment

# Editor report: Recommendation: Spotlight on the trophic response of crop plants under shade temporal fluctuation — R1/PR10

**DOI:** 10.1017/qpb.2026.10049.pr10

**Affiliations:** Biology, University of York, United Kingdom of Great Britain and Northern Ireland

No accompanying comment.

# Editor report: Decision: Spotlight on the trophic response of crop plants under shade temporal fluctuation — R1/PR11

**DOI:** 10.1017/qpb.2026.10049.pr11

**Affiliations:** RDP, ENS de Lyon, France

No accompanying comment.
